# Variability of Multiple Sclerosis Walking Scale and Multiple Sclerosis Impact Scale Scores in People Without Multiple Sclerosis

**DOI:** 10.7759/cureus.51811

**Published:** 2024-01-07

**Authors:** Tarub Binshalan, Ellen Buckley, Siva Nair, Alisdair Mcneill

**Affiliations:** 1 Neuroscience, The University of Sheffield, Sheffield, GBR; 2 Neurology, Sheffield Teaching Hospitals NHS Foundation Trust, Sheffield, GBR

**Keywords:** physiotherapy, clinical trial, mobility, quality of life, multiple sclerosis

## Abstract

Introduction

Many people with multiple sclerosis (pwMS) experience problems with mobility at some point in their disease course. The Multiple Sclerosis Impact Scale (MSIS) and Multiple Sclerosis Walking Scale (MSWS) are validated patient-reported outcome measures of physical impairment in pwMS. The range of scores on MSIS and MSWS in people without MS (pwoMS) are not well understood.

Methods

People over the age of 16 who did not have a diagnosis of multiple sclerosis (MS) were invited to complete an online survey consisting of a general health questionnaire, MSIS and the MSWS. Scores for MSIS and MSWS from pwoMS were compared to those from a cohort of 35 pwMS from a previous study. Scores for MSIS and MSWS were correlated with age, sex and comorbidities in pwoMS.

Results

One hundred eighty-nine ambulant pwoMS were recruited (52.5% female), aged over 16 years of age. Ninety-nine percent reported no difficulty with walking, 89.4% were non-smokers, and 14% had a physical co-morbidity. None used a walking aid. For pwoMS, the MSIS score was a mean of 39.14±13.75 (range 29-127), compared to a mean of 77.2±24.94 (range 40-126) for pwMS. For pwoMS, the mean MSWS score was 8.46±16.2 (0-87) compared to a mean of 56.9±28.9 (4-100) for pwMS. There was no significant effect of sex or smoking on MSIS or MSWS scores in pwoMS. Presence of a physical co-morbidity was associated with significantly higher MSIS and MSWS scores in pwoMS. There was a significant correlation of increasing age with increasing MSWS score in pwoMS but no correlation of age with MSIS score.

Conclusion

There is a wide range of MSWS and MSIS scores in pwoMS. The age and presence of comorbidities influence both MSWS and MSIS scores. Our findings have implications for the selection of control groups for clinical studies in pwMS.

## Introduction

Multiple sclerosis (MS) is an inflammatory neurological disorder characterized by demyelination of the central nervous system, resulting in a lifelong impairment in young adults. The disease causes various neurological symptoms that affect physical, psychological, and cognitive functions in people with MS (pwMS) [1]. Capturing MS symptoms is challenging due to the heterogeneous nature of the disease over time. Therefore, several Patient Reported Outcome Measures (PROMs) have been developed over the years to assess various aspects of functioning and quality of life related to MS [2-6]. Some PROMs assess specific functions related to MS, for example, the Multiple Sclerosis Walking Scale-12 (MSWS-12) [3], while other questionnaires, such as the Multiple Sclerosis Impact Scale-29 (MSIS-29), evaluate the impact of MS on multiple aspects of the person's life.

The MSWS-12, developed by Hobart et al. (2003) [3], is a 12-item scale used to assess the impact of MS in patients’ walking in the past two weeks. Patients are asked to score how much their MS has impacted each item between Not at all (1) - Extremely (5). The total score (maximum 60) is transformed to a scale ranging from 0 to 100. A higher score indicates worse walking ability. The validity and responsiveness of MSWS-12 were verified in a community sample of 149 pwMS as well as in 53 hospital outpatients [7]. Validity was also confirmed with accelerometry in a community-based MS sample [8]. There was a strong correlation between MSWS-12 scores and oxygen cost when patients performed comfortable-paced walking [9]. Learmonth et al. (2013) [10] found acceptable reliability measures for MSWS-12 within six months. Based on the results of a large-scale EU study of 290 MS patients, MSWS-12 proved to be a superior measure (compared to the timed 25-foot walk) for detecting changes after physical therapy intervention, especially in patients with moderate to severe MS [11]. Patients considered an improvement of 8 points in MSWS-12 over a 24-week period to be meaningful [12].

The MSIS-29 measures the quality of life as perceived by patients within the two weeks prior to assessment [2]. Patients completed a total of 29 questions to score for each item how much their MS has limited or bothered them between Not at all (1) - Extremely (5). Sub-scores can be computed related to physical health (items 1-20, MSISphysical) and psychological health (items 21-29, MSISpsychological). The total scores (maximum 145) are transformed into a range from 0-100, where higher scores indicate a greater degree of disability. It has been validated for use in clinical settings [7]. Both the physical and psychological domains of MSIS-29 were found to be responsive when compared to other PROMs for people with MS; MSISphysical and MSISpsychological were first and second most effective measures [13]. Several studies have confirmed the psychometric quality of the MSIS-29, with a minimal clinically meaningful change in the physical subscale reported to be 7.5 - 8.5 points [14,15].

Naturally, the majority of the literature on MSWS-12 and MSIS-29 is for pwMS. However, it is necessary to take into account the variability in values on these PROMs within the general population [16]. Variability in MSWS-12 and MSIS-29 in people without MS (pwoMS) would indicate that medical conditions other than MS can influence scores on these PROMs. It is important to understand this, since these factors may confound analysis of MSIS-29 and MSWS-12 scores in people with MS (pwMS). Such data would provide a better understanding of results in pwMS by enabling a better understanding of the impact of non-MS conditions on the health of pwMS. Availability of the normal ranges for these PROMs would also improve clinical study design by facilitating accurate power and sample size calculations. Therefore, the purpose of this study is to collect MSWS-12 and MSIS-29 norms in pwoMS and to compare this to a cohort of pwMS. 

## Materials and methods

Participant recruitment

Participants were recruited from people without a diagnosis of neurological disease at the University of Sheffield. The inclusion criteria were age over 16 years and being able to provide informed consent. The exclusion criteria was diagnosis of a neurological condition. Ethical approval was obtained from the University of Sheffield (approval number 051763). Written informed consent was provided by all participants. 

Adult, ambulatory pwMS were previously recruited to an interventional study of Remote Ischaemic Preconditioning (NCT03967106). Ethical approval was obtained from Yorkshire & The Humber - Bradford Leeds Research Ethics Committee (REC reference 19/YH/0187). These patients provided written informed consent including consent for their data collected to be used in future research.

Data collection

For pwoMS, data was collected via an online survey using Google Forms. Participants completed demographic information (age, sex, smoking status) and presence and type of comorbidity. Adapted MSIS-29 and MSWS-12 were also collected via Google Forms where reference to “MS” was removed from the questionnaire prompt for this cohort.

For pwMS, age and sex data was collected through study proforma as well as details regarding MS subtype, disease duration and Expanded Disability Status Scale (EDSS) step score. MSIS-29 and MSWS-12 were completed through paper forms and extracted from baseline (pre-randomisation) dataset. Smoking status and comorbidity details were not available in this cohort for comparison.

Statistical analysis

All analyses were performed using PASW (IBM statistics). PROMs scores were compared between pwoMS with and without co-morbidities using a Wilcoxon-signed rank test (with Bonferroni correction). PROMs scores were compared between pwoMS who did and did not smoke using a Wilcoxon-signed rank test (with Bonferroni correction). The influence of age on PROMs scores in pwoMS was examined using a one-way ANOVA. A post-hoc analysis using the Scheffe test was performed to identify differences between age levels in MSWS-12 scores. The MSWS-12 scores in pwoMS were stratified according to the ranges proposed by Goldmann et al. (2017) [17], which were associated with gait disability (MSWS-12 score 25-50), unemployment (50-75) and inability to perform activities of daily living (75-100) in pwMS.

Data availability

Anonymised data for MSIS-29 and MSWS-12 are available from the authors upon reasonable request. The data are not publicly available due to other ongoing analyses and publications.

## Results

Demographic details for both cohorts are summarised in Table 1. A total of 198 individuals without MS (104/98, 52% female) participated in this study. Of these, 29 (15%) have a physical co-morbidity: 13 (6.6%) have a lung condition, six (3%) have diabetes, six (3%) have arthritis, three (1.5%) have heart disease, and one (0.5%) has kidney disease. Only 21 (10.6%) were current smokers, and two (1%) reported having difficulty walking but none reported using walking aids. 

**Table 1:** Demographic data of participants Data presented as number (percentage %) N/A, non-applicable; pwoMS, people without MS; pwMS, people with MS; COPD, chronic obstructive pulmonary disease

Variables	pwoMS (n=198)	pwMS (n=35)
Sex		
Male	94 (47.5%)	22 (62.9%)
Female	104 (52.5 %)	13 (37.1%)
Age Level		
1 (16-30)	44 (22.2%)	5 (14.3%)
2 (30-35)	48 (24.2 %)	3 (8.6%)
3 (35-40)	50 (25.3%)	3 (8.6%)
4 (40-50)	35 (17.7 %)	7 (20%)
5 (50-70+)	21 (10.6%)	17 (48.6%)
Difficulty Walking		
No	196 (99%)	N/A
Yes	2 (1%)	
Tobacco Smoking		
No	177 (89.4%)	N/A
Yes	21 (10.6%)	
Presence of Co-morbidity Diagnosis		
Arthritis	6 (3%)	N/A
Lung Condition (e.g., Asthma or COPD)	13 (6.6 %)	N/A
Diabetes	6 (3%)	N/A
Heart Condition (e.g., Ischemic Heart Disease)	3 (1.5%)	N/A
Kidney Condition (e.g., Renal Impairment)	1 (0.5%)	N/A
None	169 (85%)	N/A

Among the 35 pwMS included in this study (37% female), 21 (60%) had relapsing and remitting MS, seven (20%) had primary progressive MS, and seven (20%) had secondary progressive MS. The mean disease duration was 12 years (standard deviation eight years). On examination, the median EDSS score was 4.0 (range 1-7) and 18 (46%) pwMS had EDSS step > 4.5 which primarily indicates the presence of walking disability.

For pwoMS, no significant difference was found between smokers and non-smokers for total MSIS-29 (P = 0.95), physical subscale (P = 0.61) or psychological subscale (P = 0.28). Smoking did not affect MSWS-12 scores (P = 0.77). However, there was a significant difference between people with and without a physical comorbidity on the MSIS-29 (P<0.001), MSISphysical (P = 0.003) and MSISpsychological (P<0.001) as well as the MSWS-12 (P<0.001) (Table 2). The difference in MSIS-29 total and subscores and MSWS-12 scores according to the participants' comorbidity is detailed in Table 3.

**Table 2:** PROMs difference within the pwoMS group Data presented as mean±SD. Wilcoxon signed-rank test. Significance level threshold after Bonferroni correction p<0.0125. MSWS-12, multiple sclerosis walking score-12; MSIS-29, Multiple Sclerosis Impact Scale; pwoMS, people without MS; PROMs, Patient Reported Outcome Measures

PROMS	Smoking status		
	Smokers (n=21)	Non-smokers (n= 177)	p value
MSIS-29	39.9±14.9	39.05±13.45	p=0.950
MSIS_Physical_	8.75 ± 13.9	6.62 ± 10.95	p=0.610
MSIS_Psychological_	10.84 ± 16.5	13.19 ± 16.24	p=0.289
MSWS-12	7.14 ± 15.08	8.99 ± 16.04	p=0.771
	Co-morbidity		
	Diseased (n=29)	Non-diseased (n=169)	p value
MSIS-29	45.75 ± 15.08	38.0 ± 13.01	p<0.001
MSIS_Physical_	11.29 ± 13.39	6.08 ± 10.73	p=0.003
MSIS_Psychological_	21.45 ± 17.53	11.48 ± 15.6	p<0.001
MSWS-12	20.21 ± 17.78	6.81 ± 14.74	p<0.001

**Table 3:** MSIS-29 and MSWS-12 scores of participants with comorbidity Only one person has a kidney condition. Data presented as Mean ± SD MSWS-12, multiple sclerosis walking score-12; MSIS-29, Multiple Sclerosis Impact Scale

	MSIS-29	MSIS_Physical_	MSIS_Psychological _	MSWS-12
Arthritis (n=6)	42.16 ± 13.77	9.17 ± 15.13	16.2 ± 19.11	21.8 ± 20.61
Lung condition (n=13)	49.2 ± 18.3	13.65 ± 15.7	25.85 ± 19.97	16.35 ± 12.81
Diabetes (n=6)	46.83 ± 12.85	11.25 ± 10.9	24.3 ± 13.99	17.95 ± 16.6
Heart condition (n=3)	39.67 ± 6.65	9.2 ± 6.4	9.3 ± 4.24	45.0 ± 21.07

Comparing the effect of age on MSIS-29 and MSWS-12 in pwoMS (Table 4), there were no statistically significant differences in the MSIS-29 (F=0.86, P = 0.491), MSISphysical (F=1.27, P =0.282) and MSISpsychological (F=1.41, P = 0.23). MSWS-12 showed a significant increase in score based on age (F=5.95, P<0.001).

**Table 4:** PROMs difference within pwoMS based on age level using one-way ANOVA Data presented as mean +/- SD.  Significance taken at p<0.05. MSWS-12, multiple sclerosis walking score-12; MSIS-29, Multiple Sclerosis Impact Scale; pwoMS, people without MS; PROMs, Patient Reported Outcome Measures

	16-30 (n=44)	30-35 (n=48)	35-40 (n=50)	40-50 (n=35)	50-70+ (n=21)	
MSIS-29	40.63±12.07	36.62±7.58	38.46±14.59	41.57±20.63	39.33±9.48	F=0.856, p=0.491
MSIS_Physical_	6.56±8.06	4.24±5.21	7.15±12.85	9.71±17.63	7.91±9.13	F=1.27, p=0.282
MSIS_Psychological _	17.73±19.85	11.74±12.79	10.38±14.7	13.33±20.06	11.11±8.87	F=1.41, p=0.23
MSWS-12	4.89±9.07	5.32±8.64	7.41±18.48	12.2±21.07	22.15±16.08	F=5.95, p=<0.001

Using the post-hoc Scheffe test for multiple comparisons, we found that the mean value of MSWS-12 was significantly higher in the age group 50-70+ compared to younger age groups (Table 5). A significant difference was found between the age groups 50-70+ and 16-30 (mean difference = 17.26, P = 0.002), between age groups 50-70+ and 30-35 (mean difference = 16.83, P = 0.002) and between age groups 50-70+ and 35-40 (mean difference = 14.74, P = 0.009). There was no significant association between age and MSIS-29. However, there was a trend towards an increase in psychological MSIS scores in the younger group. While for the MSISphysical, there was an inconsistent association of score with increased age. 

**Table 5:** Post-hoc multiple comparison of MSWS-12 scores and age level Mean difference calculated as age group 2-1 and associated p value. The mean level is significant at the p=0.01 level. MSWS-12, multiple sclerosis walking score-12

1→ 2↓	30-35	35-40	40-50	50-70+
16-30	0.425, p=1.000	2.52, p=0.958	7.31, p=0.346	17.26, p=0.002
30-35	---	2.09, p=0.977	6.89, p=0.390	16.83, p=0.002
35-40	---	---	4.79, p=0.724	14.74, p=0.009
40-50	---	---	---	9.94, p=0.232

**Figure 1:**
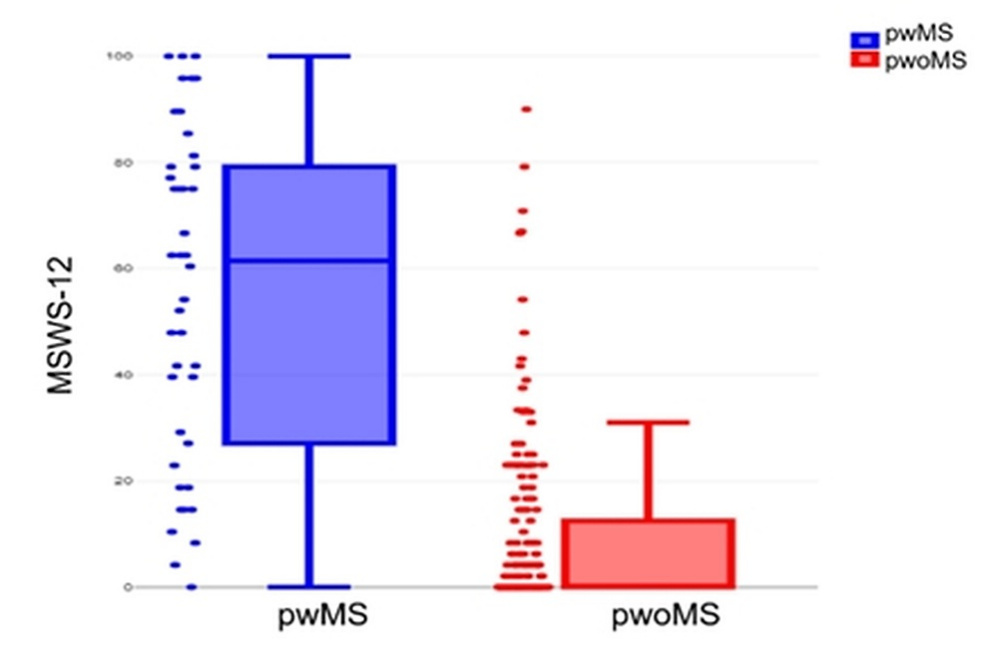
MSWS-12 score presented as median (horizontal line) and interquartile range (upper and lower edges of box) MSWS-12, multiple sclerosis walking score-12; pwMS, people with MS; pwoMS, people without MS

**Figure 2:**
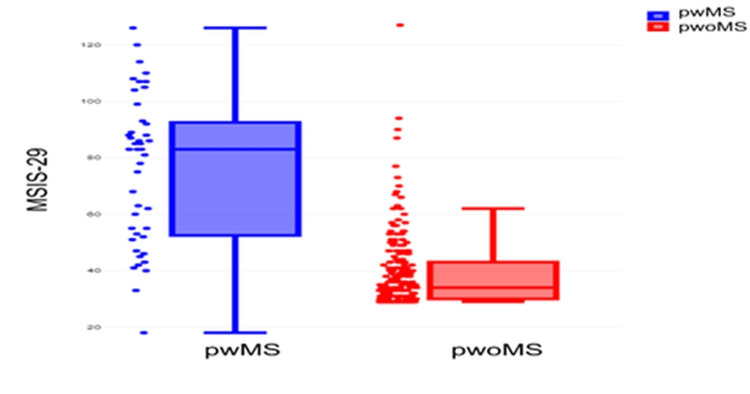
MSIS-29 score presented as median (horizontal line) and interquartile range (upper and lower edges of box) MSIS-29, Multiple Sclerosis Impact Scale; pwMS, people with MS; pwoMS, people without MS

Significant differences were observed in PROMs scores between pwoMS and pwMS (Table 6). MSIS-29 overall scores were significantly higher in pwMS (P<0.001) as well as MSISphysical (P<0.001) and MSISpsychological (P<0.001). There was also a significant difference in MSWS-12 between pwoMS individuals and pwMS (P<0.001). Distribution of MSIS-29 and MSWS-12 scores across cohorts are displayed in Figures 1 and 2. The within-group analysis of physical and psychological subscales, however, found no significant differences in the pwMS (P=0.484). Whereas for pwoMS, this difference was significant (P<0.001) with higher psychological than physical subscale scores for MSIS-29. 

**Table 6:** Comparison of PROMs scores between pwMS and pwoMS Data presented as mean ± SD; Significance level after Bonferroni correction (p<0.0125) MSWS-12, multiple sclerosis walking score-12; MSIS-29, Multiple Sclerosis Impact Scale; pwMS, people with MS; pwoMS, people without MS; PROMs, Patient Reported Outcome Measures

	pwoMS (n = 198)	pwMS (n = 35)	P Value
MSIS-29	39.14±13.57	77.2±24.94	<0.001
MSIS_Physical_	6.8±11.28	42.1±23.14	<0.001
MSIS_Psychological_	12.95±16.24	45.55±29.23	<0.001
MSWS-12	8.46±16.2	56.9±28.9	<0.001
Comparison of MSIS-29, MSIS_Physical_ & MSIS_psychological_ P value	<0.001	0.48	

There was overlap of MSWS-12 scores between pwoMS and pwMS. Fifteen pwoMS scored in the MSWS-12 range associated with gait disability (25-50). Four pwoMS scored in the 50-75 range on MSWS-12, which is associated with unemployment in pwMS. Two pwoMS scored in the MSWS-12 range 75-100; which is associated with inability to perform activities of daily living. 

Table 7 shows that there were no significant differences between males and females in either pwoMS or pwMS with regard to the MSIS-29, the MSISphysical, the MSISpsychological or MSWS-12.

**Table 7:** PROMs difference between pwoMS and pwMS based on sex Data presented as mean±SD. Significance level after Bonferroni correction (p<0.0125) MSWS-12, multiple sclerosis walking score-12; MSIS-29, Multiple Sclerosis Impact Scale; pwMS, people with MS; pwoMS, people without MS; PROMs, Patient Reported Outcome Measures

PROMS	sex	pwoMS n =198	pwMS n=35	Between groups P value
MSIS-29	Female Male	41.2±16.02 36.77±9.8	76.5±26.35 78.38±23.34	<0.001 <0.001
	p value	0.019	0.833	
MSIS_Physical_	Female Male	8.4±13.49 5.1±7.9	38.69±24.52 47.88±20.17	<0.001 <0.001
	p value	0.043	0.262	
MSIS_Psychological_	Female Male	15.46±17.98 10.16±13.6	47.47±31.75 42.30±25.28	<0.001 <0.001
	p value	0.021	0.62	
MSWS-12	Female Male	10.4±18.8 6.3±12.4	53.13±27.70 63.23+30.94	<0.001 <0.001
	p value	0.081	0.32	

## Discussion

MSIS-29 and MSWS-12 are frequently used both in observational studies and clinical trials in pwMS. Therefore, understanding the range of scores for these PROMs in pwoMS, with a range of ages and co-morbidities, can inform selection of appropriately matched control groups in clinical trials and observational studies. The purpose of this study was to document the range of scores of the MSIS-29 and MSWS-12 among pwoMS and to provide a comparison against a cohort of pwMS. This study explores the impact of age, sex, and co-morbid non-MS conditions on MSIS-29 and MSWS-12. By recruiting a varied cohort of pwoMS rather than relying on an idealised “healthy” control cohort, we provide insight into factors influencing MSIS-29 and MSWS-12 responses and how these differ from pwMS.

Matching for age and sex is an accepted principle when recruiting control groups. Understanding the effect of these variables on MSIS-29 and MSWS-12 in pwoMS is therefore crucial. In this cohort, for pwoMS, there was no significant difference between males and females for MSIS-29 total or sub scores, or MSWS-12 total scores. Increasing age was not associated with MSIS-29 total scores, but was associated with increased MSWS-12 scores. Post-hoc analysis revealed that this was driven by higher MSWS-12 scores in the 50-70-year-old age group. Our results are in keeping with findings from other studies of mobility in the general population. Yamada et al. (2021) [18] identified that every year of ageing is associated with an increased chance of mobility impairment, this was most marked in people over the age of 40 years old. This is in line with our findings demonstrating the highest MSWS-12 scores in the oldest age group of pwoMS. Yamada et al. also reported that physical co-morbidity (diabetes mellitus, hypertension, stroke, heart disease, arthritis) increases the risk of mobility impairment in the general population. A history of stroke or orthopaedic surgery had the strongest association with decreased mobility. Our sample size was too small to investigate the relationship between MSWS-12 scores and different co-morbidities. However, the highest MSWS-12 scores were seen in people with heart disease in our cohort. 

Neurological conditions such as MS impact mobility and ability to complete activities of daily life. Therefore, self-reported measures such as MSIS-29 and MSWS-12 scores are useful to capture impact of their condition on the items discussed. However, the impact of non-neurological comorbidities on MSIS-29 and MSWS-12 has not been documented. Participants from the pwoMS group who reported any physical comorbidity scored significantly less well on both the MSIS-29 (total, physical and psychological sub-scales) and MSWS-12. There is a large body of evidence documenting how cardio-respiratory disease and arthritis can impair real-world mobility performance [19-21]. It is therefore unsurprising that MSWS-12 and MSIS-29 scores are higher in pwoMS who have these co-morbidities. The psychological subscale of MSIS-29 was also significantly higher in pwoMS who had a co-morbid condition. This is in keeping with studies demonstrating a higher prevalence of conditions such as anxiety and depression in people with chronic illnesses [22]. Smoking status had no impact on MSIS-29 or MSWS-12 scores. This may reflect a lack of sensitivity for MSWS-12 and MSIS-29 to detect any subtle decrease in mobility associated with the cardio-respiratory effects of smoking. 

Overall pwMS scored significantly higher in MSIS-29 and MSWS-12 scores than in the pwoMS cohort. This corresponds to the EDSS scores in this cohort, which indicate presence of walking gait disability. Meanwhile, for pwoMS only 1% of participants reported difficulty walking and 85.7% scored in the unimpaired range for mobility MSWS-12. Although there was overlap in scores for both MSIS-29 and MSWS-12 for the pwoMS and the pwMS group, threshold scores indicate that a larger proportion of the pwMS reported impaired mobility [17]. Mobility disability and therefore MSWS-12 scores can be impacted by comorbidities alongside MS diagnosis, therefore further study is required to establish cross correlation between mobility in pwMS in the presence of comorbidities. Also, while the Goldman thresholds have been included here for reference, additional work is required to ascertain if there are similar associations in pwoMS between MSWS-12 score and daily activities.

Our study has several limitations. Our sample size did not enable us to statistically analyse the effect of different physical co-morbidities on MSWS-12 or MSIS-29 scores in pwoMS. For practical reasons, we only asked for participants to report a limited range of co-morbidities, therefore it was not possible to explore the influence of other co-morbidities (in particular psychiatric conditions or cognitive impairment) on MSWS-12 or MSIS-29. Due to the online nature of our survey, we were unable to obtain data on participants’ body mass index (BMI). Since high BMI can be a risk factor for mobility disability [23] this is an important area for future study in this population. We also did not capture data on exercise frequency, ethnicity or medication usage, all of which could influence scores on the selected PROMs. It would be useful to capture longitudinal data on MSWS-12 and MSIS-29 scores in pwoMS, to demonstrate the reliability of these PROMs in pwoMS. Lastly, it should be acknowledged that MSIS-29 and MSWS-12 were validated for use in pwMS, and it cannot be assumed that elevated scores on these PROMs from pwoMS have the same clinical significance as in pwMS. 

## Conclusions

In conclusion, we report the range of scores for MSIS-29 and MSWS-12 in pwoMS, and the factors influencing the scores on these PROMs in pwoMS. Our findings have implications for the design of clinical studies. In pwoMS, increasing age is associated with increased MSWS-12 scores. Careful age matching between cases and controls in studies is therefore vital, to prevent confounding. Physical comorbidities (e.g., cardio-respiratory disease, arthritis) are associated with high scores on MSIS-29 and MSWS-12. The presence of these in control groups must be carefully considered, since the presence of comorbidities in the control group could result in elevated MSIS-29 or MSWS-12 scores which confound statistical analyses. Likewise, the presence of physical co-morbidities in pwMS can result in increased scores on MSIS-29 and MSWS-12 which are not related to disability from MS. In clinical studies using these PROMs, cases and control groups should be matched for non-neurological co-morbidity. Given the prevalence of elevated scores on MSIS-29 and MSWS-12 in pwoMS, future work might investigate the profile of scores of other PROMs utilised in clinical studies of MS in pwoMS. 
